# Mitochondrial dysfunction in obesity: potential benefit and mechanism of Co-enzyme Q10 supplementation in metabolic syndrome

**DOI:** 10.1186/2251-6581-13-60

**Published:** 2014-05-23

**Authors:** Md Ashraful Alam, Md Mahbubur Rahman

**Affiliations:** 1 School of Biomedical Science, The University of Queensland, Brisbane, Australia; 2 Department of Pharmaceutical Sciences, North South University, Dhaka, Bangladesh

**Keywords:** Metabolic syndrome, Co-enzyme Q10, Obesity, Inflammation, Oxidative stress

## Abstract

Co-enzyme Q10 (Co-Q10) is an essential component of the mitochondrial electron transport chain. Most cells are sensitive to co-enzyme Q10 (Co-Q10) deficiency. This deficiency has been implicated in several clinical disorders such as heart failure, hypertension, Parkinson’s disease and obesity. The lipid lowering drug statin inhibits conversion of HMG-CoA to mevalonate and lowers plasma Co-Q10 concentrations. However, supplementation with Co-Q10 improves the pathophysiological condition of statin therapy. Recent evidence suggests that Co-Q10 supplementation may be useful for the treatment of obesity, oxidative stress and the inflammatory process in metabolic syndrome. The anti-inflammatory response and lipid metabolizing effect of Co-Q10 is probably mediated by transcriptional regulation of inflammation and lipid metabolism. This paper reviews the evidence showing beneficial role of Co-Q10 supplementation and its potential mechanism of action on contributing factors of metabolic and cardiovascular complications.

## Introduction

**Figure 1:**
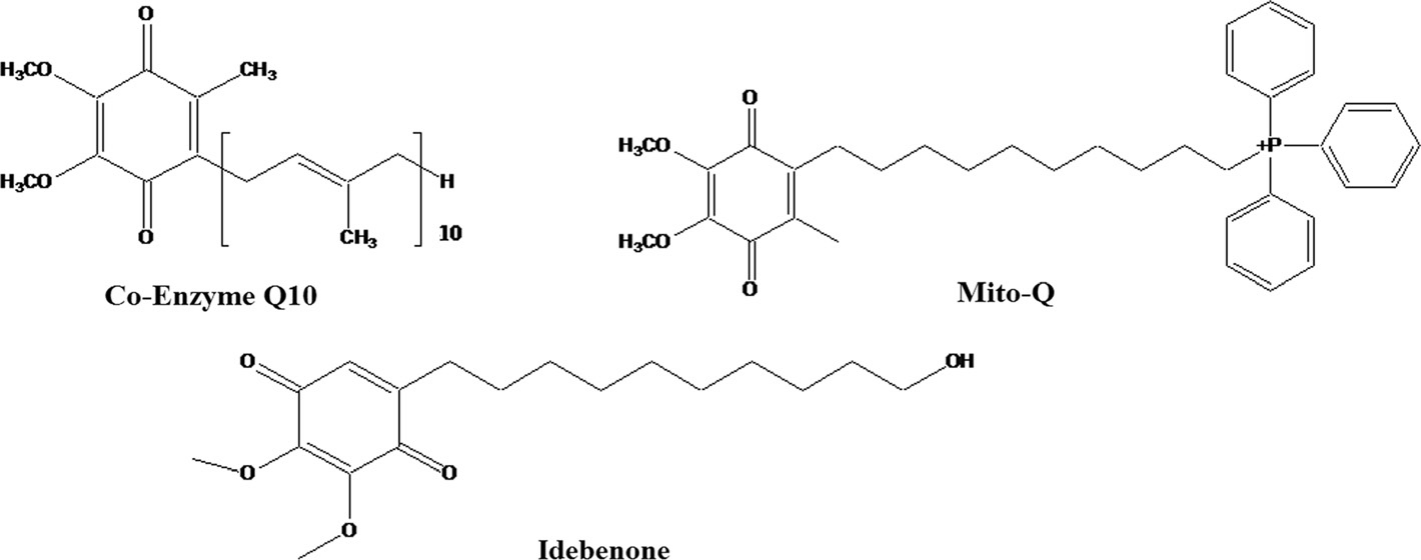
Schematic diagram of Co-Q10, Mito-Q and Idebenone.

Metabolic syndrome is a cluster of disease symptoms such as dyslipidemia, hyperglycemia and insulin resistance, hypertension and visceral obesity [1]. Oxidative stress and inflammation are pivotal in all stages of atherosclerosis, hypertension, and non-alcoholic fatty liver and in subjects with metabolic syndrome [2,3]. Mitochondrial dysfunction plays a crucial role in the development of diabetes and metabolic disorder [4]. Both, animal and clinical studies revealed that the sources of free radicals would be from mitochondrial origin [4]. The mitochondrial electron transport chain is the generator of free radicals mainly singlet oxygen (O^.-^) while producing the ATP from the substrate molecule. Free radicals may react with other important molecules within cells and enhance lipid peroxidation, oxidize proteins and damage DNA and are thus responsible for oxidative stress [2]. Inflammatory responses are also responsible for most of the organ dysfunction in metabolic disorder. Increased concentration and expression of tumor necrosis factor-α (TNF-α), interleukin-6 (IL-6), and monocyte chemoattractant protein-1 (MCP-1) are evident in adipocyte dysfunction and insulin resistance in obesity and metabolic syndrome [5]. Furthermore, inflammatory cells infiltration are also increased in adipose tissues which is responsible for adipocyte dysfunction [6]. Inflammation in adipose tissues could be a causative factor diminishing mitochondrial biogenesis and energy homoeostasis [7-9]. Therefore, supplementation with a dietary antioxidant having anti-inflammatory properties would be beneficial which can scavenge free radicals and restores antioxidant defence as well as suppresses inflammatory responses. Co-enzyme Q10 (Co-Q10), an integral part of the mitochondrial electron transport chain which transports electrons and acts as a natural antioxidant (Figure 1). Co-Q10 can also be found in much of the human diet. Beneficial effects of Co-Q10 supplementation have been noted for most of the symptoms of metabolic syndrome, e.g. hypertension, diabetes, liver diseases, insulin resistance and obesity. This review will thus, focus on the effect of Co-Q10 on various component of metabolic syndrome and elucidate its potential mechanism of action.

### Origin, chemistry and absorption of Co-Q10

Chemically Co-Q10 is a quinone molecule found almost in all cells of the body, hence the term ubiquinones [10]. It exists in nature and in the body, ubiquinones, the oxidized form and ubiquinol, the reduced form. Co-Q10 serves as an essential carrier for the electron transfer in the mitochondrial respiratory chain for the synthesis of ATP. Co-Q10 was first isolated in 1957 from beef mitochondria [10]. Folkers et al. revealed the chemical structure of Co-Q10 in 1958 [11]. Meat, poultry and fish are the richest sources of Co-Q10, and the daily intake of these foods provides between 3 to 5 mg of Co-Q10 [12,13]. The absorption of Co-Q10 from the diet occurs mainly in the small intestine and is better absorbed in presence of lipid rich foods [14]. Co-Q10 is then transported to the liver and form lipoprotein complex and deposited in tissues [14]. Tissues with high-energy requirements and metabolic rates such as the heart and the skeletal muscle contain relatively high concentrations of Co-Q10 [15]. About 95% of Co-Q10 in human circulatory system exists in its reduced form as ubiquinol [16]. The safety of high doses of orally-ingested Co-Q10 over long periods is well documented in human [17] and also in animals [18]. Co-Q10 dosages generally range from 100 to 200 mg a day for patients suffering cardiovascular disease [19].

### Mitochondrial biogenesis and Co-Q10

Mitochondrial biogenesis means the increased number and function of mitochondria due to the response of increased cellular energy demand and thereby increased ATP production. Generally, mitochondrial electron transport chain consists of several protein complexes, namely, complex I, complex II, complex III and complex IV etc. [20]. NADH which donates electrons to complex I and FADH_2_ feed the electron to complex II. Glucose oxidation through glycolysis and pyruvate dehydrogenase to acetyl CoA generates NADH. In contrast, fatty acid oxidation to acetyl-CoA generates FADH_2_ through the process of β-oxidation. Acetyl CoA from both sources feeds the TCA cycle. Electrons coming from NADH and FADH_2_ are thus transported via reduced Co-Q10 to Complex III [20]. The proton gradient is generated as a consequence of this sequential process, drives complex V or the ATPase to produce ATP. ATP is thus transported via ANP to its target site. In presence of uncoupling agent, the protomotive force will divert to UCPs and a proton leak occurs. Thus the energy will expend as heat without producing any ATP. Complex I and complex III are the primary sources of O_2_ free radicals due to incomplete reduction of the oxygen molecule. The Mitochondrial environment maintains a highly protective defence against this free radical. In normal physiological conditions these free radicals are scavenged by superoxide dismutase of mitochondrial origin and later by catalase in cytosol. Diabetic hyperglycemia and metabolic disorder are responsible for the inactivity of these antioxidant enzyme systems and decreased mitochondrial function. Several evidences suggest that, inhibition of the mitochondrial electron transport chain activity may increase lipid accumulation in adipocyte [21,22].

Mitochondrial biogenesis can be modulated by several transcriptional regulators present in the cell. Peroxisome proliferator activated receptor (PPAR) family is such a regulator of mitochondrial biogenesis [23]. Three types of PPAR proteins have been identified so far, PPAR-α, PPAR-γ and PPAR-δ [24]. PPARs activation is also important for lipid metabolism, adipocyte differentiation and the prevention of inflammation [24]. Moreover, PPARs also regulate mitochondrial biogenesis via an activator called peroxisome proliferator-activated receptor gamma coactivator-1α (PGC-1α) [25,26]. PGC-1α is physiologically regulated by exercise [27,28] and calorie restriction [29]. Apart from these exercise and calorie restriction means, pharmacological agents such as fenofibrates [30] and resveratrol [31] stimulate PGC-1α and restore mitochondrial function. Co-Q10 mediated activation of PPARs are revealed only recently which would be a possible mechanism of energy homeostasis in failing tissues and obesity [32].

### Metabolic disorder due to Co-Q10 deficiency

Ogashara et al. described the first patients (two sisters) with primary Co-Q10 deficiency in 1989 [33]. They had progressive muscle weakness, abnormal fatigue, and central nervous system dysfunction from early childhood characterized by a low Co-Q10 concentration in their muscles. Both patients improved remarkably with oral Co-Q10 [33]. Maintaining adequate Co-Q10 level throughout the body is important for normal function and health. Plasma concentrations of Co-Q10 are high in healthy infant and children and declining with age [34,35]. Metabolic disorder may arise due to Co-Q10 deficiency. Growing bodies of evidences indicate that oxidative stress plays a critical role in the pathogenesis of type 2 diabetes mellitus and its complications [36]. Low plasma Co-Q10 concentrations were found in patients with poor glycaemic control and diabetic complications [37-40].

HMG-CoA reductase inhibitors (statins) reduce Co-Q10 levels in human [41,42]. Alternatively, supplementation with oral Co-Q10 can restore plasma Co-Q10 levels in patients receiving statin therapy [41-43]. Statin mediated Co-Q10 depletion affects muscle function. Patients taking statin to reduce plasma lipids suffered myalgia and myopathic pain [44-46]. Myocardial depletion of Co-Q10 has also been demonstrated in heart failure patients with cardiomyopathy which was improved by Co-Q10 therapy [47]. An important factor contributing to statin related myopathy may be a genetic susceptibility to muscle disorders and underlying metabolic mechanisms [48,49]. Due to the statin therapy, genomic variation has been found in *COQ2* gene which encoding para-hydroxybenzoate-polyprenyl transferase for CoQ10 biosynthesis [48]. Another report suggests that statins may affect energy metabolism (Carnitine palmitoyltransferase II deficiency) combined with a genetic susceptibility triggering myopathic outcomes in certain high-risk patients [49].

### Antioxidant effect of Co-Q10

Benzoquinone group of Co-Q10 is able to accept and donate electrons which is a critical feature for an antioxidant [14]. It scavenges free radicals and inhibits lipid and protein peroxidation. Vitamin E and Co-Q10 prevents lipid peroxidation at nearly the same rate [50]. However, Co-Q10 prevents LDL oxidation more efficiently than α-tocopherol, lycopene, or β-carotene [51]. Co-Q10 also enhances the availability of other antioxidants such as vitamin C, vitamin E and beta-carotene [52]. Direct elimination of free radical such as lipid peroxyl, peroxyl and/or alkoxyl radicals *in vitro* and *in vivo* as a consequence of Co-Q10 supplementation was reported by several investigators [53,54]. Co-Q10 may also serve as an antioxidant by acting as a cofactor and activator of mitochondrial uncoupling proteins, leading to a reduction in free radical generation *in vivo*[55]. H_2_O_2_-induced DNA strand breaks in lymphocytes are protected by ubiquinol-10 [56]. In another study, *in vivo* supplementation with Co-Q10 was shown to enhance the recovery of human lymphocytes from oxidative DNA damage [57]. Improved cellular antioxidant enzyme activity was also noted for Co-Q10 supplementation. Co-Q10 treatment increased antioxidant enzyme activity of superoxide dismutase, catalase, and glutathione in the liver homogenates of diabetic rats followed by reduced lipid peroxidation [58]. Antioxidant enzyme, catalase activity and GSH concentration were also improved in liver of acetaminophen induced rats [59]. A recent clinical study also showed that Co-Q10 supplementation at a dose of 150 mg decreased oxidative stress and improved antioxidant enzyme activity in patients with coronary artery disease [60].

### Effect of Co-Q10 on inflammation and metabolic syndrome

Inflammation is a response to tissue or organ damage from exogenous and endogenous factors and assists in the restoration of impaired homeostasis. Several inflammatory cytokine are also generated e.g. interleukin-1 (IL-1), interleukin-6 (IL 6), tumour necrosis factor- α (TNF-α) etc. which have both systemic and local effect. Local effects are associated with increased expression of adhesion cell molecules such as intracellular adhesion molecule-1 (ICAM-1), selectins and heat-shock proteins [61]. Macrophage infiltration and fibroblast activation are two inflammatory responses develop chronically in inflamed adipose tissues [6,62]. Systemic chronic inflammatory response is also considered to be a mediator of metabolic syndrome and insulin resistance [62,63]. Pro-inflammatory cytokines and oxidative stress have been shown to be responsible for developing metabolic disturbances, such as insulin resistance and activation of immune response in liver, adipose tissue and in muscle [64-66]. Moreover, activation of inflammatory pathways in hepatocytes is sufficient to cause both local as well as systemic insulin resistance [67,68]. Recent studies have confirmed positive association between obesity indices and inflammatory markers, mainly c-reactive protein (CRP) and other inflammatory cytokines [69-71]. Evidence is also starting to accumulate that inflammatory cytokines are over expressed in adipose tissues of obese rodent models and obese humans [72-75]. Some other studies suggest that systemic administration of the TNF-α also induces insulin resistance in experimental animal [76,77]. In recent years, several inflammatory signal mechanisms have been described such as c-JUN kinase (JNK) pathways, protein kinase-C (PKC) and IκB kinase (IKK) mediated nuclear factor- κB (NF-κB) pathways and suppressor of cytokine signaling (SOCS) family mediated pathways [63]. Furthermore, PKC and IKK can be activated by increased fatty acid level in cells [63].

**Figure 2:**
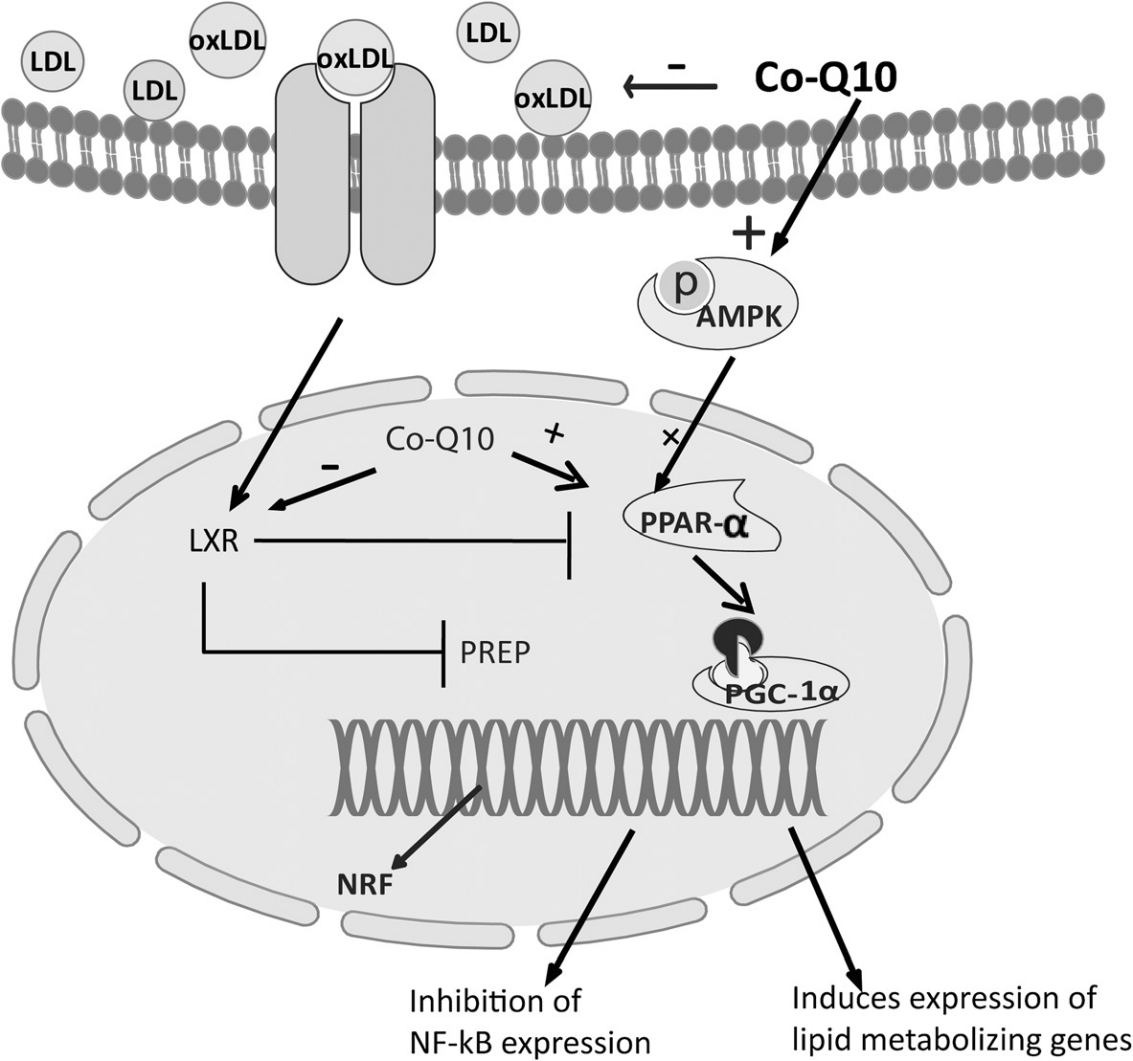
**Proposed mechanism of Co-Q supplementation on anti-inflammatory and lipid metabolism pathways in tissues in case of metabolic syndrome.** AMPK, Adenosine monophosphate activated protein kinase; PPAR, Peroxisome proliferator activated receptor; PGC-1α, Peroxisome proliferator-activated receptor gamma coactivator-1; oxLDL, Oxidized Low density lipoprotein; NRF, nuclear respiration factor; LXR, Liver X receptor; PPRE PPAR response element.

Low density lipoprotein (LDL) cholesterol oxidation is the key regulator for developing inflammation in endothelial cells and other tissues [78]. The LDL receptor plays a vital role in increasing uptake of cholesterol from plasma to cell and increasing clearance of apoB and apoE-containing lipoproteins [79]. In diabetes and obesity, LDL-R populations decrease and increase LDL level in plasma [80]. LDLR-/- mice showed an increased plasma lipid profile and an increase in inflammatory markers in response to high fat diet [79,81,82]. The anti-atherogenic effect of PPARγ agonist was seen in LDLR-/- male mice which is correlated with improved insulin sensitivity and decreased tissue expression of TNF-α and gelatinase B [83]. However, peroxisome proliferator activated receptor-γ (PPAR-γ) is reported to attenuate inflammation in activated macrophages by interfering with NF-κB signalling [84]. PPAR-α is another analogue of the PPAR family also regulates anti-inflammatory genes (Figure 2) [85].

The anti-inflammatory activity of Co-Q10 is well documented. Stimulation of cells with LPS resulted in a distinct release of TNF-α, macrophage inflammatory protein-1 alpha (MIP-1α) and monocyte chemo attractant protein-1 (MCP-1) which were significantly attenuated by pre-incubation of cells with the reduced form of Co-Q10 [86,87]. Treatment with CoQ10 also reduced the elevated plasma lipid profiles and decreased mRNA expression of the pro-inflammatory cytokine TNF-α in adipose tissues of *ob*/*ob* mice [88]. CoQ10 supplementation also improved the inflammatory state in liver of high fat fructose diet fed rats [89]. Recent evidence suggests that Co-Q10 can serve as an agonist of PPARs and activates the PPAR mediated anti-inflammatory response (Figure 2) [90,91].

### Effect of Co-Q10 on endothelial dysfunction and hypertension

Endothelial dysfunction and hypertension are common in metabolic syndrome. Increased glucose intolerance and oxidative stress plays a critical role in the development of endothelial dysfunction in aortas of diabetic rats [36]. Co-Q10 supplementation improved the endothelial dysfunction in the mesenteric arteries in high fat diet fed SHRsp rats [92]. Inflammatory cell infiltration may be a potential mediator of inflammation and endothelial dysfunction in aortas. Co-Q10 prevented the lymphocytic infiltration in perivascular area of aortas from fructose fed rats [93]. Co-Q10 also improved endothelial dysfunction in statin-treated type II diabetic patients [94]. Considerable evidence indicates that oxLDL-induced endothelial dysfunction is associated with down-regulation of eNOS and up-regulation of inducible nitric oxide synthase (iNOS). A recent study showed that Co-Q10 prevented apoptosis in human umbilical vein endothelial cells (HUVEC) due to oxLDL by preventing the NF-kB mediated caspase-3 activation [95]. Co-Q10 also attenuated the oxLDL-mediated down-regulation of endothelial nitric oxide synthase (eNOS) and up-regulation of inducible nitric oxide synthase (iNOS) [95]. oxLDL induced inflammatory process and expression of adhesion molecules, release of pro-inflammatory cytokines and the adherence of monocyte were also attenuated by CoQ10 in THP-1 cells [95]. Extracellular superoxide dismutase (ecSOD) activity and endothelium-dependent vasodilatation were also improved remarkably after CoQ10 supplementation which alters local vascular oxidative stress [96].

The antihypertensive effect of Co-Q10 is also well documented in animal and human studies. Co-Q10 supplementation reduced hypertension and cardiac hypertrophy in DOCA-salt hypertensive rats [97,98]. The Co-Q10 analogue decylubiquinone (10 mg/kg) reduced the systolic blood pressure, plasma malondialdehyde, total cholesterol and LDL-cholesterol in the SHRsp rats [99]. A recent meta-analysis of clinical trials investigating the use of Co-Q10 for treatment of hypertension considered 12 trials since 1975 and found beneficial effect of Co-Q10 supplementation [100]. Among these trials, four were prospective randomized trials and eight trials considering the effect of Co-Q10 on final blood pressure compared with previous level [100]. Co-Q10 supplementation has shown to be effective in lowering blood pressure in diabetes, improves the glycaemia control in metabolic syndrome; however, a recent study showed that Co-Q10 treatment failed to reduce blood pressure in patients with uncontrolled hypertension [101]. Another study suggests that treatment with Co-Q10 (50 mg twice a day for ten weeks) in patients with essential hypertension reduced hypertension without affecting the plasma renin activity, serum and urinary sodium and potassium, and urinary aldosterone [102]. These results suggest that treatment with Co-Q10 decreases blood pressure in patients with essential hypertension, possibly because of a reduction in peripheral resistance [102]. However, the exact mechanism is not known, but one theory proposed that it reduces peripheral resistance by preserving nitric oxide bioavailability [103]. Alternatively, coenzyme Q10 may increase the synthesis and sensitivity of prostacyclin (PGI_2_), a potent vasodilator and inhibitor of platelet aggregation, to arterial smooth muscle and does relaxation to arteries [104].

### Effect of Co-Q10 on cardiac dysfunction

Clinically, Co-Q10 has potential for the prevention and treatment of cardiovascular diseases such as myocardial infarction, congestive heart failure and other drug- induced/disease induced cardiomyopathies [105]. The heart is highly sensitive to Co-Q10 deficiency. Defective levels of specific oxidative phosphorylation/respiratory enzyme activities and reduced energy reserve in heart failure may be considered as contributing factors for the progression of disease [106]. Low levels of Co-Q10 concentration were found in 70–75% of patients with aortic stenosis or insufficiency, mitral stenosis or insufficiency, diabetic cardiomyopathy, atrial septal defects and ventricular septal defects [107]. Circulating levels of Co-Q10 were also significantly lower in patients with ischemic heart disease and in patients with dilated cardiomyopathy as compared to healthy controls [108]. Pepe et al. have reviewed the meta analysis of clinical trials of Co-Q10 in heart failure, reporting that cardiac output, cardiac index and stroke volumes were improved with the treatment of Co-Q10 [103]. Diastolic dysfunction is one of the earliest identifiable signs of myocardial failure due to severe thickening of the left ventricles, which accounts for 30-49% of heart failure cases. Patients treated with 200 mg/day of Co-Q10 improved interventricular septal thickness significantly with improved symptoms of fatigue and dyspnea with no side effects noted [109]. Studies on isolated rat’s heart also demonstrated a protective effect of Co-Q10 against ischemia and reperfusion injuries [110,111]. Pre-treatment with Co-Q10 improved recovery of cardiac function, aerobic efficiency and enzyme levels in young healthy rats compared to untreated controls undergone experimentally induced ischemia-reperfusion in heart [112]. In an another study, mice pre-treated with Co-Q10 for 4 days prior to toxic doses of adriamycin, survival rates were significantly higher (80%) compared to the mice not received the supplements (40%) [113]. This Co-Q10 mediated protection against adriamycin induced cardiac toxicities is probably due to the inhibition of lipid peroxidation and induction antioxidant enzymes in cardiac tissue [114].

### Effect of Co-Q10 on diabetes and insulin resistance

Coenzyme Q concentrations appear to be reduced in diabetic states. Diabetic animals showed decreased Co-Q10 concentration in heart, liver and skeletal muscle [115]. Evidence also exists for reduced Co-Q in plasma of humans with diabetes. Lower plasma levels of CoQH_2_ have been found in diabetic patients than healthy subjects [37,116]. Supplementation with Co-Q10 would be beneficial for diabetes and insulin resistance. Co-Q10 supplementation (10 mg/kg) improved the elevated glucose concentration and glucose intolerance in STZ induced diabetic rats without affecting the insulin concentration [58]. However, 200 mg of Co-Q10 daily for 6 months did not improve glycemic control or serum lipid levels of Type-2 diabetics [117]. In another randomized double-blind, placebo-controlled study, Co-Q10 supplementation at a dose 100 mg for three months failed to improve HbA1c, mean daily blood glucose concentrations, mean insulin dose, number of hypoglycemic episodes or cholesterol concentrations compared to the placebo group [118]. Improved glycemic control due to Co-Q10 supplementation could be a protective action against pancreatic beta cell destruction in diabetes. Co-Q10 supplementation did not reduce the pancreatic damage, inflammation and beta cell loss in diabetic rats but decrease glycated HbA1c and pancreatic lipid peroxidation [119]. Further researches are required to evaluate the hypoglycaemic action of Co-Q10 supplementation.

### Liver steatosis and effect of Co-Q10 on hepatic dysfunction

Oxidative stress and inflammatory responses are responsible for hepatic damage and fibrosis. The liver X receptors (LXR) constitute a class of nuclear receptors activated by oxidized lipids (Figure 2). Activation of LXR in macrophages induces expression of several genes involved in lipid metabolism and reverse cholesterol transport [120]. Activation of these transcription factors inhibits inflammatory gene expression in macrophages and adipocytes, largely through PPAR-γ mediated suppression of NF-κB signalling [121,122]. LXR also plays an important role in lipid and cholesterol metabolism. LXRα knockout mice develop enlarged fatty livers, degeneration of liver cells, high cholesterol levels in liver, and impaired liver function when fed a high-cholesterol diet [123]. Generally, oxidized LDL serves as an agonist of LXR and modulates PPAR binding to DNA sequence elements termed peroxisome proliferator response elements (PPRE) [124]. Thus, antioxidants such as Co-Q10 may prevent LDL oxidation and serve as an LXR antagonist. Recent report showed that reduced form of Co-Q10 downregulated genes involved in cholesterol biosynthesis (HMGCS1, HMGCL and HMGCR) which are also deactivated by transcriptional regulators PPARα and LXR/RXR complex in liver of SAMP1 mice [125]. However, oxidized form of Co-Q10 did not alter the genes expression of HMGCS1, HMGCL and HMGCR [91,125]. A recent report also suggests that LXRs influence CoQ synthesis without directly regulating the process and CO-Q10 concentration was found to be decreased in liver of LXR-α knockout mice and LXR double knockout mice [126].

A high fat diet and fructose feeding can also cause dyslipidemia and hepatic steatosis. Co-Q10 supplementation increases life-span of rats fed a diet enriched with polyunsaturated fatty acids [127,128]. High fat diet induced hepatic oxidative stress in rats was improved by supplementation with Co-Q10 followed by the improvement of the plasma lipid profile [89]. The lipid lowering effect of Co-Q10 supplementation was also seen in fructose fed rats [93]. A Co-Q10 analogue, Q monomethyl ether, also showed beneficial effect on progressive non-alcoholic fatty liver (NAFL) in rats fed a high fat diet and improved liver architecture by preventing the fat droplet accumulation in hepatocytes [129]. Increased fatty acid beta oxidation would be the potential mechanism of improving lipid profile in metabolic syndrome. Scanty literature was found on any effect of Co-Q10 on mitochondrial fatty acid beta oxidation. However, up regulation of the beta oxidation gene was observed in animal treated with ubiquinol [90]. Recent evidence suggests that this up-regulation of fatty acid oxidation is probably involved PPAR mediated pathways [90]. It is now evident that ubiquinol, a reduced form of Co-Q10, may be an activator of PPAR gene expression and activated a series of lipid metabolizing gene family in mice [90,91]. Alternatively, ubiquinol downregulated a series of genes involved in cholesterol and fatty acid synthesis such as HMGCS1, HMGCL and HMGCR [91]. These genes are also negatively regulated by PPAR mediated pathways.

Co-Q10 supplementation also showed hepatic protection in other model of hepatic dysfunction. Co-Q10 treatment showed hepatic protection against acetaminophen induced liver toxicities in rats [59]. Co-enzyme Q10 treatment improved extensive centrilobular necrosis, cytoplasmic vacuolization and ballooning degeneration of hepatocytes with congested sinusoids in liver of rats [59]. Hepatic protection in these rats depends on the blocking NF-kB mediated inflammatory signal pathways and inactivation of caspase activity [59]. Co-Q10 supplementation is also effective in preventing the toxin induced hepatic damage [130]. Co-Q10 supplementation in diet also showed hepatic protection in aged rats due to enhanced cellular antioxidant action [131].

### Effect of Co-Q10 on obesity and fat metabolism

Generally, obese individuals showed a reduction of fat metabolism and increased fat deposition in the body. Increased fat deposition is responsible for the hyperglycemia, insulin resistance, dyslipidemia and hypertension, most of the common features of metabolic syndrome [132]. Mitochondrial dysfunction and reduced Co-Q10 concentration was found in obese individual [133]. Adipocyte differentiation and fat deposition into adipocytes play important roles in obesity. Co-Q10 prevents adipogenesis in rosiglitazone induced adipogenesis in ob/ob mice [88]. Anti-adipogenic activity of Co-Q10 was also shown in 3 T3-F442A cell line. Inhibition of Co-Q10 synthesis strongly triggered adipocyte differentiation while increment of Co-Q 10 synthesis strongly inhibited adipocyte differentiation [134]. Co-Q10 treatment increases fat oxidation and energy expenditure in inguinal white adipose tissue. Decreased mRNA expression of the lipogenic enzymes fatty acid synthase (FAS) and acetyl-CoA carboxylase 1 (ACC1), and the glycerogenic enzyme phosphoenolpyruvate carboxykinase (PEPCK) are responsible for the lipid lowering effect of Co-Q10 [88]. In this study, mRNA expression of proteins involved in mitochondrial biogenesis (peroxisome proliferator-activated receptor-g coactivator-1 (PGC-1)), oxidative phosphorylation system (OXPHOS) system (cytochrome oxidase subunit IV (COIV)), fatty acid transport into mitochondria (carnitine-palmitoyl transferase 1, muscle isoform (M-CPT1)) and energy expenditure (uncoupling protein-1 (UCP1)) were also increased to promote the fatty acid utilization in high fat diet fed mice [88]. AMPK regulates the expression of lipogenic genes, including fatty acid synthesis (FAS) [135]. Co-Q10 increased the AMPK phosphorylation in 3 T3-L1preadipocytes probably by increasing the cytoplasmic calcium concentrations followed by increased Ca^2+^/calmodulin-dependent protein kinase kinase (CaMKK). Moreover, Co-Q10 increased fatty acid oxidation in 3 T3-L1preadipocytes and increased PPARα in protein and mRNA level [32]. This AMPK-mediated PPARα induction at least in part causes suppression of adipocyte differentiation [32].

### Future perspectives and conclusion

Co-Q10 has proven potential as an antioxidant molecule with anti-inflammatory properties. Recent evidence also suggests that Co-Q10 may serve as AMPK and PPARs activators and increases the fat burning capacity of cells (Table 1). Its use is still limited due to poor water solubility and lipophilic nature. Several analogues have so far been synthesized such as mito-Q and idebenone.

**Table 1:** Effect of Co-Q10 supplementation on lipid metabolism in metabolic syndrome

**Parameter**	**Model and dose**	**Effect and potential mechanism**	**Reference**
**Lipid metabolism**	3 T3-L1 pre-adipocytes	-Increases fatty acid beta oxidation.	[32]
		- ↑ Ca^++^ Influx; ↑AMPK; ↑PPAR-α.	
		-Prevents adipocytes differentiation	
	Fructose fed rat	-↓Total cholesterol; ↓LDL-Cholesterol; ↓triglycerides	[93]
	ob/ob mice	- ↓ Total cholesterol; ↓triglycerides; ↓NEFA	[88]
		- ↓ mRNA expression of the lipogenic enzymes such as fatty acid synthase (FAS) and acetyl-CoA carboxylase 1 (ACC1).	
	C57BL6J mice	- Increases fatty acid beta oxidation	[90]

Mito-Q10 comprises a lipophilic triphenylphosphonium cation covalently attached to an ubiquinol antioxidant [136]. The lipophilic triphenylphosphonium cation helps this molecule to easily cross the lipid bilayer, be orally bio-available and accumulate in mitochondria several hundred fold compared with Co-Q10 itself [136]. MitoQ10 has been shown to be effective against mitochondrial oxidative damage in vivo and in rodent models of sepsis and reperfusion injury of heart [137,138]. Administration of MitoQ10 also protects against the development of hypertension, improves endothelial function, and reduces cardiac hypertrophy in young stroke-prone spontaneously hypertensive rats [139]. MitoQ, was shown to completely prevent mitochondrial abnormalities as well as cardiac dysfunction characterized by a diastolic dysfunction [140]. Recent evidence suggests that Mito-Q is effective against ethanol induced micro and macro hepatic steatosis in rats [141]. Metabolic dysfunctions were also improved in mice treated with Mito-Q. Mito-Q supplementation reduced the fat mass and plasma lipid profile in ApoE^-/-^ mice [142]. MitoQ supplementation also improved hyperglycemia, hepatic steatosis and decreased DNA oxidative damage (8-oxo-G) in multiple organs of ApoE^-/-^ mice [142]. Idebenone, a benzoquinone carrying exactly the same quinone moiety as Co-Q0, Co-Q1 and Co-Q10, shows multiple activities *in vitro* and *in vivo*. Idebenone is quickly absorbed and is well tolerated and safe given as single or repeated daily doses [143]. Like Co-Q10, idebenone also prevents lipid peroxidation and ROS production *in vivo*[144-146]. However, to date, no more literature has been found with an idebenone effect on diabetes and metabolic syndrome. In view of the above discussion, Co-Q10 supplementation has proven its efficacy and benefit to treat metabolic syndrome and obesity. Further research is warranted to get benefit in a clinical setup.

## Abbreviations

AMPK: Adenosine monophosphate activated protein kinase; ATP: Adenosine triphosphate; CAT: Catalase; ERK: Extracellular receptor kinase; GPx: Glutathione peroxidase; GST: Glutathione S-transferase; HUVEC: Human umbilical vein endothelial cell; IL-6: Interleukin 6; IRS: Insulin receptor substrate; LDL: Low density lipoprotein; LXR: Liver X receptor; MAPK: Mitogen activated protein kinase; MCP-1: Monocyte chemotactic protein-1; mtTFA: Mitochondrial transcription factor A; NO: Nitric oxide; NRF: Nuclear respiration factor; PGC-1α: Peroxisome proliferator-activated receptor gamma coactivator-1α; PPAR: Peroxisome proliferator activated receptor; PPRE: PPAR response element; ROS: Reactive oxygen substrate; SOD: Superoxide dismutase; STZ: Streptozotocine; TNF: Tumour necrosis factor; VSMC: Vascular smooth muscle cell; oxLDL: Oxidized low density lipoprotein; VCAM-1: Vascular cell adhesion molecule-1; UCP-1: Uncoupling protein- 1.

## Competing interest

The authors declare that they do not have any competing interests regarding this manuscript.

## Authors’ contribution

MAA generated the idea, literature search for the review and drafted the manuscript. MMR prepared the drawings, figures and drafted the manuscript. Both authors read and approved the final manuscript.
